# Confinement Effects on the Magnetic Ionic Liquid 1-Ethyl-3-methylimidazolium Tetrachloroferrate(III)

**DOI:** 10.3390/molecules27175591

**Published:** 2022-08-30

**Authors:** Christopher M. Burba, Hai-Chou Chang

**Affiliations:** 1 Department of Natural Sciences, Northeastern State University, Tahlequah, OK 74464, USA; 2 Department of Chemistry, National Dong Hwa University, Shoufeng, Hualien 974, Taiwan

**Keywords:** ionic liquid, confinement, mesoporous silicas, magnetic materials, vibrational spectroscopy

## Abstract

Confinement effects for the magnetoresponsive ionic liquid 1-ethyl-3-methylimidazolium tetrachloroferrate(III), [C_2_mim]FeCl_4_, are explored from thermal, spectroscopic, and magnetic points of view. Placing the ionic liquid inside SBA-15 mesoporous silica produces a significant impact on the material’s response to temperature, pressure, and magnetic fields. Isobaric thermal experiments show melting point reductions that depend on the pore diameter of the mesopores. The confinement-induced reductions in phase transition temperature follow the Gibbs–Thomson equation if a 1.60 nm non-freezable interfacial layer is postulated to exist along the pore wall. Isothermal pressure-dependent infrared spectroscopy reveals a similar modification to phase transition pressures, with the confined ionic liquid requiring higher pressures to trigger phase transformation than the unconfined system. Confinement also impedes ion transport as activation energies are elevated when the ionic liquid is placed inside the mesopores. Finally, the antiferromagnetic ordering that characterizes unconfined [C_2_mim]FeCl_4_ is suppressed when the ionic liquid is confined in 5.39-nm pores. Thus, confinement provides another avenue for manipulating the magnetic properties of this compound.

## 1. Introduction

Although ionic liquids (ILs) have been known for over a century, significant interest in these materials began in earnest only within the last twenty years [1]. The field has since blossomed into a robust class of materials with numerous applications, including energy storage, reaction solvent media, heat transfer fluids, tribology, and pharmaceuticals. These research thrusts are driven by the unique combinations of properties ILs afford, which are difficult—if not impossible—to create with “traditional” molecular liquids. For example, ILs with high ionic conductivity, low viscosity, negligible vapor pressures, and combustion resistance can be achieved through the judicious selection of cations and anions. Moreover, synthetic versatility allows molecular structures to be crafted to meet the demands of specific technological niches.

An interesting subclass of ILs possess cations or anions with unpaired electrons and, therefore, are able to interact with external magnetic fields. Archetypal magnetic ILs (MILs) are those that contain the tetrachloroferrate(III) anion [2,3,4], wherein the iron atom exists in a high spin state with five unpaired electrons [5]. Other ligand types (such as bromide) or different transition metals have also been examined [6,7,8,9,10]. These materials can undergo magnetic ordering at low temperatures, and a number of groups have sought to correlate magnetic interaction pathways to the molecular arrangements of ions in the solid phases [11,12,13,14,15,16]. For instance, de Pedro et al. [11] discovered antiferromagnetic ordering below a 3.8 K Néel temperature in 1-ethyl-3-methylimidazolium tetrachloroferrate(III) (hereafter, [C_2_mim]FeCl_4_). The magnetic properties of this MIL are temperature [11] and pressure [12] sensitive. García-Saiz et al. [12] further claimed the magnetic ordering is mediated by Fe–Cl···Cl–Fe interactions.

The inherent magnetic susceptibility of MILs opens the door for applications that are unavailable to diamagnetic ILs [17]. For example, MILs may be separated from diamagnetic components simply by placing the mixture in the vicinity of a sufficiently strong magnet [18,19,20]. Alternatively, magnetic fields can enhance gas absorption rates in a MIL. Jiang et al. [21] demonstrated this effect by exploiting the paramagnetic nature of 1-butyl-3-methylimidazolium tetrachloroferrate(III), [C_4_mim]FeCl_4_, to enhance the rate of benzene gas uptake in a magnetically-driven rotational reactor. MILs also offer interesting opportunities for gas transport, separation, and sequestration. For instance, the trajectory of gas bubbles flowing through [C_4_mim]FeCl_4_ [22] and CO_2_ permeability in a poly(vinylidene fluoride)-supported MIL [23] are both influenced by external magnetic fields. Beyond fluid-phase separations and transport, MILs have found numerous applications in catalysis [24,25,26,27,28,29,30,31,32,33], as solvents [34,35,36], supporting reaction media [37,38,39,40], electrochromic devices [41,42], and batteries [43,44,45].

The direct modification of IL molecular structure is a well-established avenue for manipulating IL properties toward desired ends. However, indirect approaches, such as confining ILs within the pore network of mesoporous or nanoporous solids, have also been pursued [46,47]. The high surface areas of the porous materials provide numerous IL-surface interaction sites that trigger new properties, which are nonexistent in the pure liquid phase, to emerge. For example, a defining feature of ILs is the presence of an extended charge alternation network that permeates the liquid phase. This complex structure appears to be converted into ion layers near solid surfaces [48]. Surface force apparatus measurements suggest ion layers persist at considerable distances from the solid–liquid interface (e.g., ion layering of 1-butyl-3-methylimidazolium bis(trifluoromethanesulfonyl)imide extends ~8 nm from atomically smooth mica) [49]. ILs can also experience structural ordering along the surface in response to the underlying substrate material [50]. Conductive interfaces, such as carbon nanotubes, profoundly impact ion–ion interactions of confined ILs [51]. Ions situated near the pore walls are able to interact with their image charges on the surface. This enables the IL to break Coulombic ordering and form co-ion pairs if the pore size is small enough to only accommodate a single IL layer. In addition to structural modification, confinement effects on IL melting points [52,53], cation–anion correlation distances [54], dynamics [55,56,57], and even magnetic properties [58,59,60,61] are all widely attested (and debated) in the literature. Ordered arrays of cylindrical SiO_2_ mesopores (e.g., MCM-41, SBA-15, and MSU-H) are the most common materials used to confine ILs. Therefore, it is possible that these surface effects could extend to the pore centers if sufficiently small pore diameters are used, suppressing any bulk-like regions of IL.

Our goal is to investigate the prototypical MIL [C_2_mim]FeCl_4_ when it is confined within mesoporous SBA-15 silicas. We then characterize the composite samples from thermal, magnetic, and spectroscopic points of view to elucidate how [C_2_mim]FeCl_4_ properties change as the pore diameter of the confining host is varied.

## 2. Results and Discussion

### 2.1. Characterization of the Mesoporous Silicas

**Figure 1:**
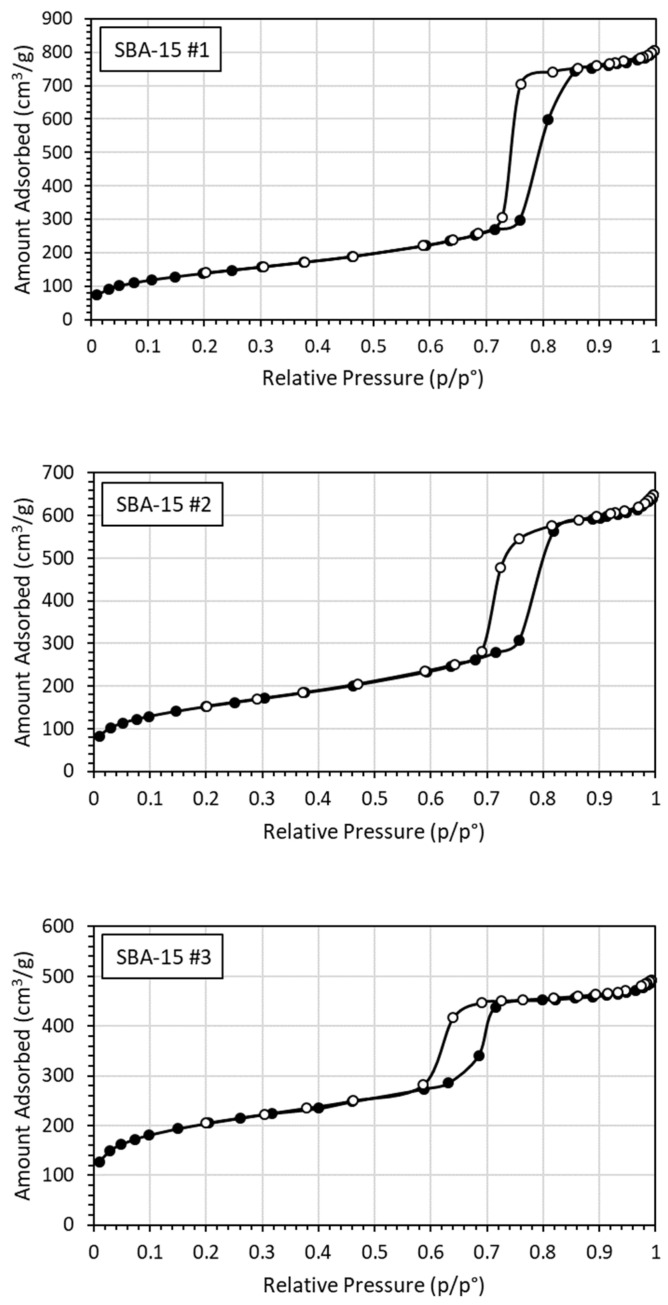
Nitrogen adsorption isotherms for SBA-15 mesoporous silica. Filled and open symbols represent the adsorption and desorption phases of the measurement, respectively.

Nitrogen physisorption isotherms for three SBA-15 silicas are presented in Figure 1. All three materials exhibit H1 hysteresis loops as defined by the IUPAC classification scheme [62,63]. This phenomenon is indicative of capillary condensation of the adsorbate within cylindrical pores and is present whenever the pore diameter is greater than ~4 nm [64]. Following the recommendation of Sing and Williams [63], BJH pore diameters are computed from the desorption isotherm.

The resulting data are presented in Table 1, along with BET surface areas of the mesoporous silicas. Pore diameters are smaller than the nominal sizes quoted by the vendor and range from 5.39 to 8.36 nm. Concomitantly, the pore volumes available to store [C_2_mim]FeCl_4_ increase from 0.67 to 1.26 cm^3^ g^−1^. Surface areas accessible to the N_2_ adsorbate decrease as the pore diameters increase.

### 2.2. Thermal Characterization

The phase behavior of the confined ionic liquid is ascertained by DSC. Thermal traces are provided in Figure S1 from Supplementary Materials; summaries of the thermal transitions are contained in Table 2. Unconfined [C_2_mim]FeCl_4_ undergoes subtle cold crystallization at 235.2 K, followed by a solid–solid phase transition at 256.8 K. The IL then melts at 287.5 K. Integration of this peak places ΔH at 63.5 J g^−1^. Constricting [C_2_mim]FeCl_4_ within SBA-15 reduces the melting point and enthalpy of fusion. The magnitude of the confinement-induced melting point reduction depends on the pore diameter of the silica host. For example, [C_2_mim]FeCl_4_ melts at 235.4 K in 5.39 nm diameter pores, whereas the melting point is 249.4 K in larger 8.36 nm diameter pores. The notable lack of an endothermic transition near 256.7 K confirms the IL is encapsulated within the pores, and the unconfined material is minimized for the composition studied.

Melting point depression is frequently observed when liquids are confined in mesoporous silica. The Gibbs–Thomson equation [65] and its extensions [66,67,68] are the most common frameworks for explaining the origin of melting point reduction. This model connects confinement-induced melting point reduction to the inverse pore radius $r^{-1}$,

(1) $$\Delta T=T_{\mathrm{confined}}-T_{\mathrm{unconfined}}=-\frac{2VT\gamma \cos\theta }{\Delta H}\left(\frac{1}{r}\right)$$

**Figure 2:**
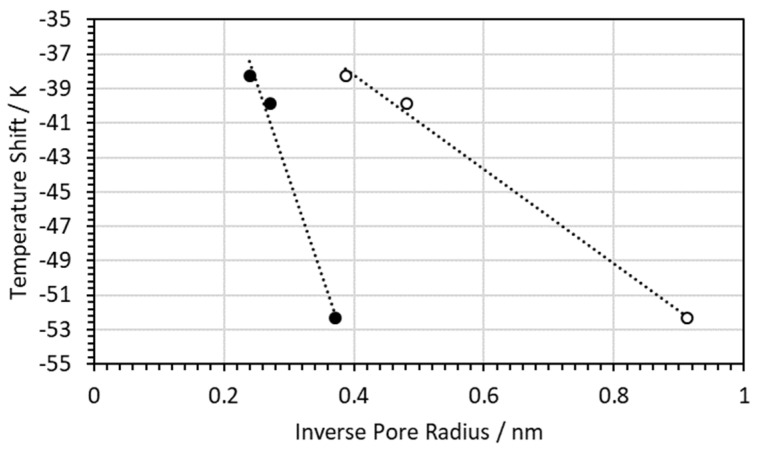
Gibbs–Thomson plots of ΔT vs. 1/r (filled circles) and ΔT vs. 1/(r−t) (open circles). Linear regressions are $\Delta T=-\frac{110.87}{r}-10.888$ with R^2^ = 0.9829 and $\Delta T=-\frac{27.384}{r-1.60}-27.236$ with R^2^ = 0.9965.

Furthermore, the magnitude of the melting point shift depends on the physical properties of the confined liquid (molar volume V, unconfined melting point temperature T, and molar enthalpy of fusion ΔH) and liquid–pore wall interfacial interactions (solid–liquid surface tension γ and contact angle θ). A Gibbs–Thomson plot for our system is provided in Figure 2, showing the predicted negative correlation between ΔT and $r^{-1}$.

It is common to modify equation 1 to include a non-freezable interfacial layer along the pore wall by replacing r with r−t, where t represents the thickness of the non-freezing shell. The presence of a non-freezable interfacial layer can explain, at least in part, the ΔH reduction when [C_2_mim]FeCl_4_ is confined in mesoporous silica. If the interfacial shell thickness is set to zero, the expected enthalpy of fusion is $\Delta H_{t=0}=\rho \pi r^{2}l\Delta \bar{H}$, where ρ is the IL density, l is the length of the pores, and $\Delta \bar{H}$ is the enthalpy of fusion divided by mass. However, t>0 will reduce the volume of ionic liquid that freezes in the pore, giving $\Delta H_{t>0}=\rho \pi {\left(r-t\right)}^{2}l\Delta \bar{H}$. Taking the ratio of the two and solving for t yields

(2) $$t=\left(1-\sqrt{\frac{\Delta H_{t>0}}{\Delta H_{t=0}}}\right)r$$

We can calculate the expected size of $\Delta H_{t=0}$ from the mass of ionic liquid trapped in the silica pores and $\Delta \bar{H}$ = 63.5 J g^−1^ from Table 2. If we set the measured ΔH for the confined liquids to $\Delta H_{t>0}$, we can solve for a non-freezable shell thickness. The analysis predicts a 1.60 ± 0.11 nm shell of [C_2_mim]FeCl_4_ that resides along the pore wall and encases the frozen MIL. Including this non-freezable interfacial layer improves the linear regression statistics for the Gibbs–Thomson plot in Figure 2: R^2^ = 0.9829 and *p* = 0.084 for t = 0 nm compared with R^2^ = 0.9965 and *p* = 0.038 for t = 1.60 nm.

Finally, the interfacial surface tension of [C_2_mim]FeCl_4_ may be approximated from the Gibbs–Thomson plot using the slope from the linear regression, the density of the MIL (1.458 g cm^−3^), enthalpy of fusion for the MIL, and the contact angle the MIL forms against the SiO_2_ wall. We measured the contact angle of the MIL on a glass slide (67° ± 3°, Figure S2 from Supplementary Materials) to estimate the contact angle [C_2_mim]FeCl_4_ forms along the pore wall. Based on these assumptions, the interfacial surface energy [C_2_mim]FeCl_4_ experiences in our SBA-15 silicas is 4.40 mJ m^−2^.

### 2.3. Impedance Spectroscopy

**Figure 3:**
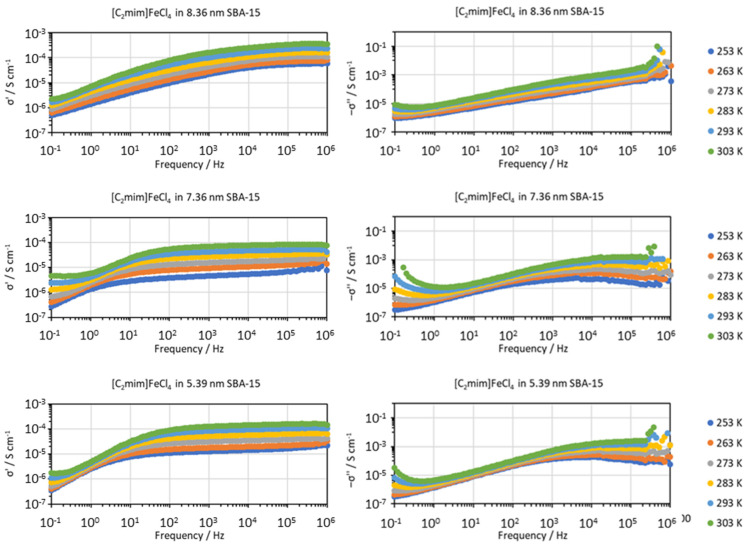
Real and imaginary parts of the complex conductivity function for [C_2_mim]FeCl_4_ confined within SBA-15 mesoporous silica.

Broadband impedance spectroscopy provides information about ion conduction when the MIL is confined. This is accomplished by converting the complex impedance function into a complex conductivity function $\sigma^{*}$. Real and imaginary parts of $\sigma^{*}$ (i.e., $\sigma^{*}=\sigma^{\prime }+i\sigma^{\prime \prime }$) are then plotted against frequency (see Figure 3).

**Figure 4:**
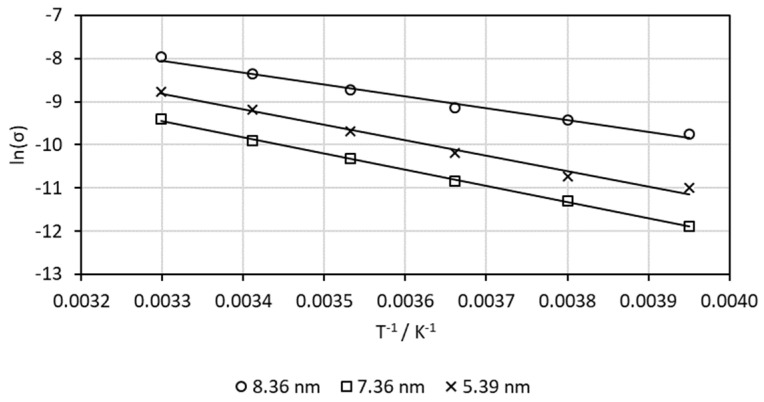
Arrhenius plots for [C_2_mim]FeCl_4_ confined in mesoporous SBA-15 silica. Conductivity units are S cm^−1^. Linear regressions are as follows: y = –2750.4x + 1.0278 (R^2^ = 0.9891) for 8.36-nm diameter pores, y = –3768.9x + 2.9906 (R^2^ = 0.9989) for 7.36-nm diameter pores, and y = –3557.1x + 2.9108 (R^2^ = 0.9885) for 5.39-nm diameter pores.

For sufficiently low applied voltages (i.e., those that fall within the linear regime), the real part of the conductivity function for an ionic liquid will display a plateau in the intermediate frequency region. The value of $\sigma^{\prime }$ along this plateau corresponds to the DC ionic conductivity. Polarization of the sample causes $\sigma^{\prime }$ to decline at lower frequencies [69]. DC ionic conductivities extracted from $\sigma^{\prime }$ are relatively similar to one another, regardless of mesopore size. For example, the 293 K conductivities are 2.3 × 10^−4^ (8.36 nm pore diameter), 5.0 × 10^−5^ (7.36 nm pore diameter), and 1.0 × 10^−4^ S cm^−1^ (5.39 nm pore diameter). By way of comparison, unconfined [C_2_mim]FeCl_4_ has a room-temperature ionic conductivity of 1.8 × 10^−2^ S cm^−1^ at 293 K [70]. The temperature dependence of the ionic conductivities is well described by the Arrhenius equation (Figure 4). The highest activation energy energies are 31.1 and 29.6 kJ mol^−1^ for [C_2_mim]FeCl_4_ in 7.36 and 5.39 nm diameter pores, respectively. A smaller activation energy of 22.9 kJ mol^−1^ is measured for the MIL in an 8.36-nm pore. All of these are higher than the activation energy for unconfined [C_2_mim]FeCl_4_ (14.9 kJ mol^−1^) [70].

Transport properties of ILs are influenced by confinement, but the response to confinement is not the same in all situations. Ionic conductivities may be enhanced [56,69] or diminished [71,72] depending on IL identity, pore wall chemical constitution, and pore loading amounts [46]. Nevertheless, it is generally recognized that ILs situated near pore walls have slower dynamics [55,73,74], and this can impact ion transport for confined ILs relative to the bulk. Fast ion conduction is thought to occur in pore centers where the IL structure most closely resembles the bulk IL [46]. Tasserit et al. [72] argue that ions experience cooperative motion when placed in confined geometries and subjected to external electric fields, and this may either facilitate or jam ion transport. The increase in [C_2_mim]FeCl_4_ activation energy upon confinement is consistent with sluggish ion transport relative to the bulk MIL. Therefore, [C_2_mim]FeCl_4_ experiences hindrances to ion transport when confined in SBA-15.

### 2.4. Magnetic Susceptibility Measurements

**Figure 5:**
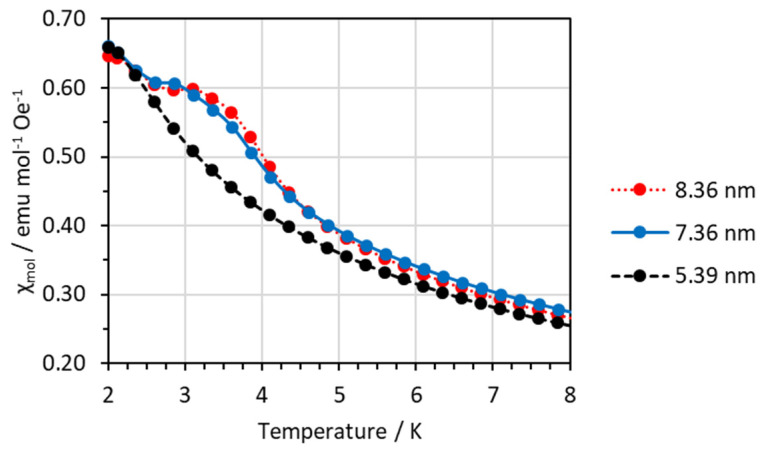
Temperature-dependent, zero-field-cooled molar magnetic susceptibilities for [C_2_mim]FeCl_4_ confined in silica 1 (red dotted line, 8.36 nm), silica 2 (blue solid line, 7.36 nm), and silica 3 (black dashed line, 5.39 nm).

**Figure 6:**
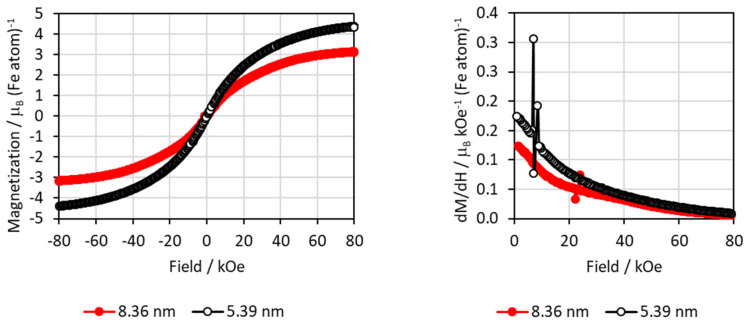
Magnetization (**left**) and first derivative (**right**) plots for [C_2_mim]FeCl_4_ confined within mesoporous SBA-15 silica.

Molar magnetic susceptibilities of confined [C_2_mim]FeCl_4_ are presented in Figure 5. Antiferromagnetic ordering is clearly observed for samples with larger pore diameters (8.36 and 7.36 nm). The ~3.6 K Néel temperature found in Figure 5 is comparable to that reported by de Pedro et al. [11] for pure [C_2_mim]FeCl_4_. However, a reduction in pore diameter to 5.39 nm eliminates the peak. Magnetization plots of zero-field cooled [C_2_mim]FeCl_4_ within mesoporous SBA-15 are provided in Figure 6. The high-spin Fe^3+^ ions found in ${\mathrm{FeCl}}_{4}^{-}$ have a theoretical value of 5 μ_B_/Fe atom. Confined MILs fail to reach the theoretical value. Inside the 5.39-nm diameter pores, the MIL reaches 4.3 μ_B_/Fe atom at 80 kOe. By way of comparison, magnetization saturation declines to 3.2 μ_B_/Fe atom when the MIL is placed in 8.36-nm diameter pores. Rates of change, dM/dH, gradually decline for both samples as a function of the applied field, H. This is different from pure IL, where magnetization rates of change were relatively constant [11]. Confined ionic liquids are known to experience significant interactions with the pore wall, including suppressed dynamics in the interfacial region, and our ion transport data suggest this confined MIL also experiences sluggish conductivities. It is possible that the slower dynamics limit spin ordering.

Otsuka and coworkers [61] also noticed suppressed antiferromagnetic ordering when 4.7-nm diameter SBA-15 is partially filled with [C_2_mim]FeCl_4_. Under these conditions, the MIL lacks a discernable Néel point in the magnetic susceptibility data, and X-ray scattering reveals increased Cl···Cl distances between adjacent anions as the temperature decreases. Based on these results, the authors concluded that the magnetic ordering of the Fe atoms on adjacent anions is inhibited when the MIL is confined. The lack of a discernable Néel point in the 5.39-nm sample presented here suggests that spin ordering is only achieved when the pore diameter is larger than 5.39 nm. As noted above, confined [C_2_mim]FeCl_4_ has a 1.60 nm non-freezable interfacial layer that forms along the pore wall. This effectively reduces the quantity of MIL that freezes to an inner core of reduced diameter. In the case of the smallest pore system studied, only the center 2.19 nm diameter region may freeze. Crystal structures of [C_2_mim]FeCl_4_ contain unit cell dimensions as large as 1.4 nm [16]. Therefore, any crystalline domains formed in the 5.39 nm pores may be quite small and unable to support the long-range antiferromagnetic ordering that characterizes the unconfined MIL. Larger-sized pores will naturally allow bigger crystalline domains to grow. Thus, the frozen [C_2_mim]FeCl_4_ in the pore interior will more closely resemble frozen [C_2_mim]FeCl_4_ when it is not confined.

### 2.5. Ambient Pressure Vibrational Spectroscopy

Infrared and Raman spectroscopy can provide information about cation–anion and ion–pore wall interactions through changes in the frequencies and relative intensities of intramolecular vibrational modes belonging to the ions. Normal mode frequencies are determined by the potential energy surface upon which the molecule resides. Changes in molecular coordination can alter the potential energy surface and cause a corresponding shift in mode frequencies. The ${\mathrm{FeCl}}_{4}^{-}$ anion belongs to the T_d_ point group; hence, its intramolecular vibrational modes are forbidden to be simultaneously IR and Raman active. Heavy Cl atoms produce relatively low-frequency vibrations (viz., <400 cm^−1^), meaning a combination of far-IR and Raman spectroscopy are required to assess the anion’s vibrational modes.

**Figure 7:**
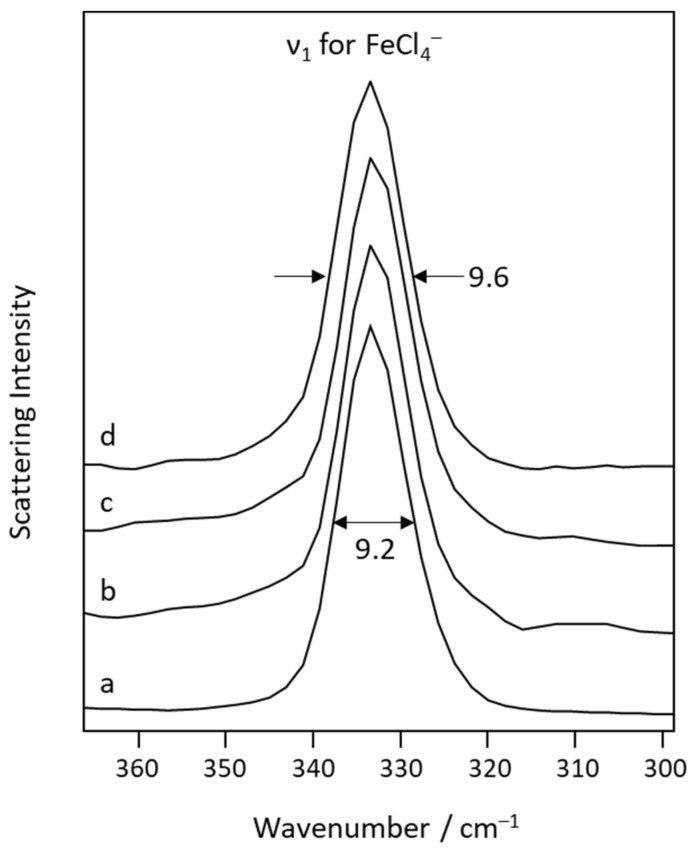
Raman spectra of (**a**) unconfined [C_2_mim]FeCl_4_ and [C_2_mim]FeCl_4_ confined within (**b**) 8.36, (**c**) 7.36, and (**d**) 5.39 nm mesoporous silica.

**Figure 8:**
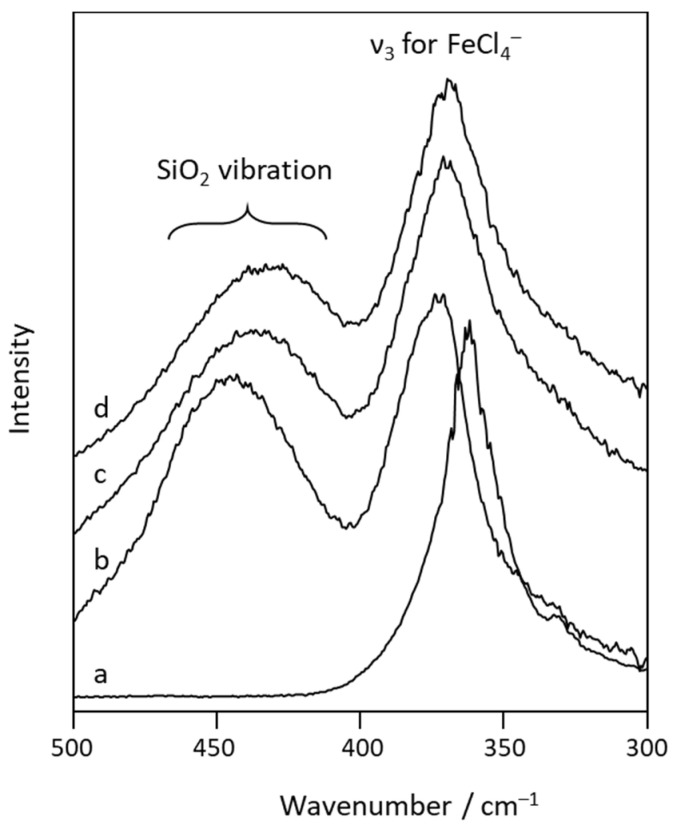
Far-IR spectra of (**a**) unconfined [C_2_mim]FeCl_4_ and [C_2_mim]FeCl_4_ confined within (**b**) 8.36, (**c**) 7.36, and (**d**) 5.39 nm mesoporous silica.

Raman spectra of confined and unconfined [C_2_mim]FeCl_4_ are displayed in Figure 7. The figure contains a single intense band at 333 cm^−1^, which is assigned to totally symmetric stretching motions of the Fe–Cl bonds. This mode is commonly referred to as ν_1_ and has A_1_ symmetry. Confining [C_2_mim]FeCl_4_ slightly broadens ν_1_ but leaves the band frequency essentially unchanged. Asymmetric Fe–Cl bond stretching motions (ν_3_) have T_2_ symmetry and are found in far-IR spectra (Figure 8). The spectra contain two overlapped bands. A broad feature centered at ~450 cm^−1^ originates from the SiO_2_ pore walls. Kirk [75] classifies amorphous SiO_2_ vibrations in terms of O atom displacements relative to two Si atoms, and this particular band is attributed to O atom rocking motions relative to an axis connecting the two adjacent Si atoms. The [C_2_mim]FeCl_4_ far-IR spectrum also contains a band at 362 cm^−1^ that we assign to ν_3_. The degeneracy of ν_3_ is preserved in the confined state, implying the symmetric solvation shell about the ${\mathrm{FeCl}}_{4}^{-}$ ion is not spoiled when the MIL is placed inside SBA-15 silicas. Interestingly, ν_3_ increases in frequency and width when the MIL is confined. These changes may originate from new molecular configurations that occur in the confined state (e.g., Si–OH···Cl–Fe interactions with the pore wall).

**Figure 9:**
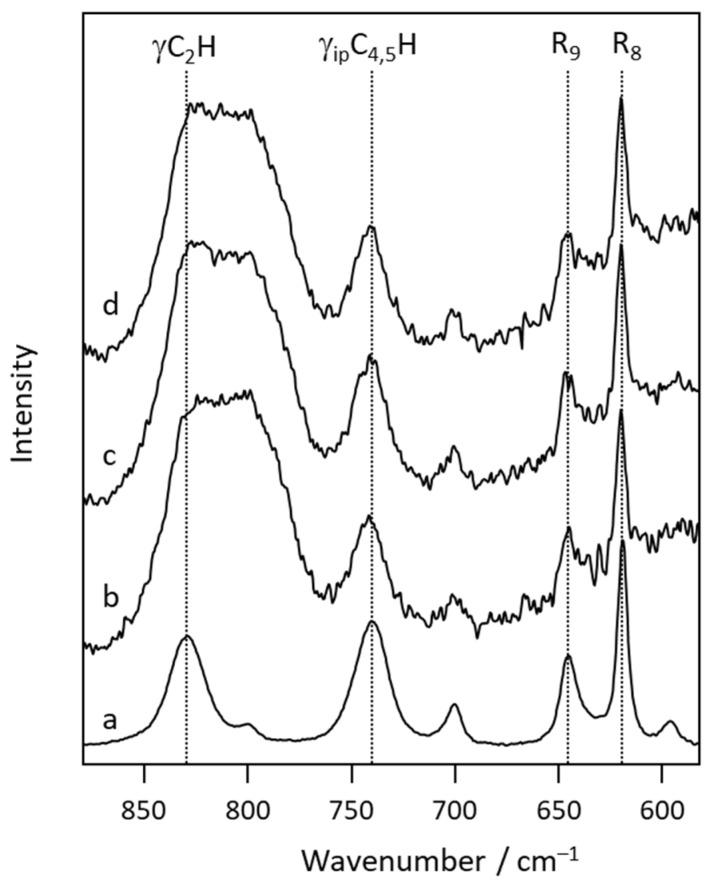
Mid-IR spectra of (**a**) unconfined [C_2_mim]FeCl_4_ and [C_2_mim]FeCl_4_ confined within (**b**) 8.36, (**c**) 7.36, and (**d**) 5.39 nm mesoporous silica.

**Scheme 1:**
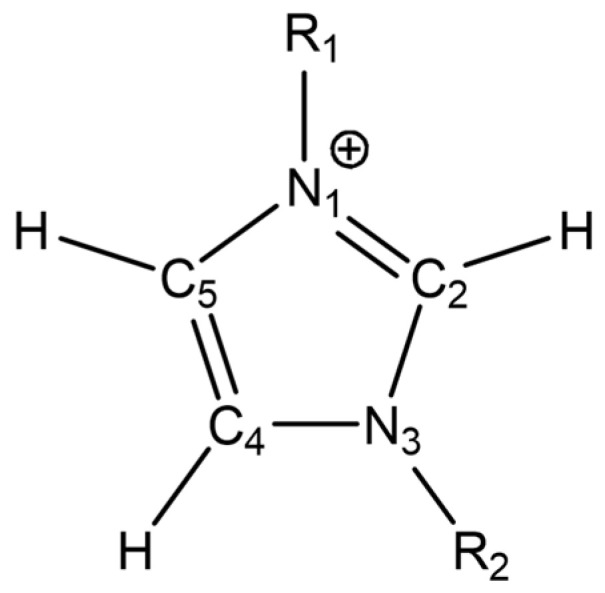
Numbering system for the imidazolium cation.

Mid-IR spectra provide information about the imidazolium cations since all anion vibrations occur below 400 cm^−1^. As expected, the spectra are dominated by bands associated with SiO_2_ vibrations from SBA-15 that overlap and obscure many of the cation bands. Fortunately, the 850 to 600 cm^−1^ region (Figure 9) contains only one relatively weak SiO_2_ band. This band, which is attributed to symmetric stretching motions of O atoms relative to Si atoms, consists of overlapped transverse optic and longitudinal optic modes at ~810 and ~820 cm^−1^, respectively [75]. Multiple bands that originate from the [C_2_mim]^+^ cations can be discerned in the IR spectra. Our band assignments follow the quantum chemical calculations and potential energy distribution analyses of Grondin et al. [76]. Normal mode eigenvectors for this ion typically involve complicated sets of atomic movements from multiple parts of a molecule. In large molecules, it is rare for a mode to neatly correspond to a single internal coordinate. This is especially true for modes having the same symmetry. Indeed, anharmonic calculations of vibrational mode coupling constants support substantial mixing of [C_2_mim]^+^ modes [77,78]. In spite of this complexity, we will refer to these vibrational modes by one or two prominent atomic motions that occur during the vibration. This is purely meant to simplify communication. We will also refer to the vibrational motions of ring-bonded H atoms according to the conventional numbering system for imidazolium ions (see Scheme 1).

Out-of-plane C–H deformations for hydrogen atoms directly bonded to the imidazolium ring occur at 830 and 740 cm^−1^. The 830 cm^−1^ band involves large C_2_–H bending motions γC_2_H, while the 740 cm^−1^ band is characterized by in-phase C_4_–H and C_5_–H bending motions γ_ip_C_4,5_H. The corresponding out-of-phase C_4_–H and C_5_–H motion has a very small dipole moment derivative and is essentially silent in the IR spectrum. The weak band near 700 cm^−1^ consists of a complicated mixture of ring atom motions. Finally, two bands at 645 and 619 cm^−1^ are assigned to R_9_ and R_8_ ring vibrations, respectively [76]. The frequencies of all these bands are essentially unchanged when the MIL is confined within the SBA-15 silicas.

### 2.6. High-Pressure Infrared Spectroscopy

**Figure 10:**
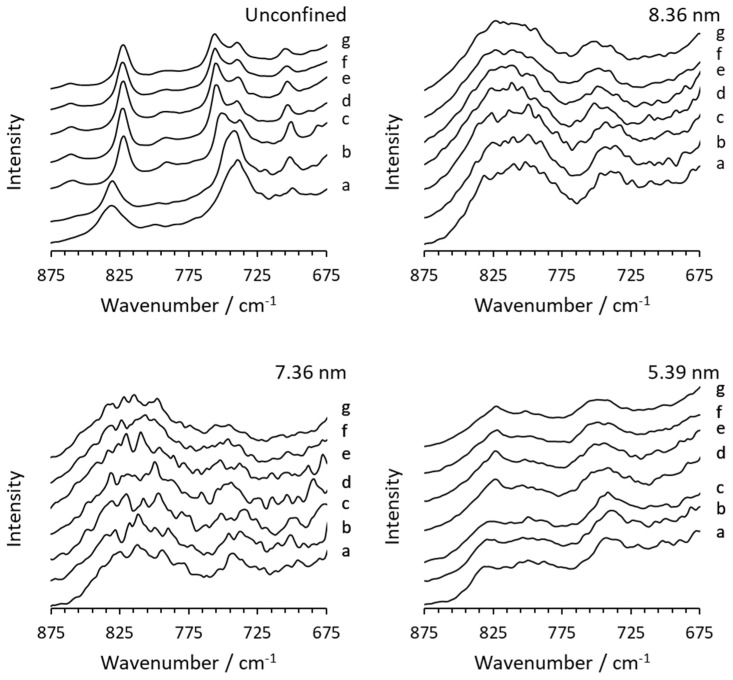
Pressure-dependent mid-IR spectra of unconfined [C_2_mim]FeCl_4_ (**upper left**) and [C_2_mim]FeCl_4_ confined within 8.36 (**upper right**), 7.36 (**lower left**), and 5.39 nm (**lower right**) mesoporous silicas. Applied pressures are (**a**) ambient, (**b**) 0.4, (**c**) 0.7, (**d**) 1.1, (**e**) 1.5, (**f**) 1.8, and (**g**) 2.5 GPa.

Pressure-dependent IR spectroscopy provides another perspective on the phase transition process for confined [C_2_mim]FeCl_4_. Mechanical forces shorten interionic distances and often trigger band frequency shifts. Phase transitions, in particular, are common in high-pressure infrared spectroscopic experiments. These are usually accompanied by band narrowing or factor group splitting [79] due to the arrangement of ions in a crystalline phase. Isothermal pressure-dependent IR spectra of unconfined [C_2_mim]FeCl_4_ are presented in Figure 10. The most striking changes occur between 0.4 and 0.7 GPa, where γC_2_H abruptly shifts from 830 to 822 cm^−1^, and the asymmetric γ_ip_C_4,5_H band centered at 742 cm^−1^ becomes two bands at 738 and 752 cm^−1^. All of these bands gradually shift to higher frequencies as the pressure is increased from 0.7 to 2.5 GPa. These spectroscopic changes indicate phase transformation occurs between 0.4 and 0.7 GPa [12,80].

Confinement modifies how the IL responds to pressure. The clearest sequence of changes appears in the spectra measured for [C_2_mim]FeCl_4_ inside 5.39 nm pores. We first note the delay of the abrupt γC_2_H red shift from 0.4–0.7 GPa (unconfined MIL) to 0.7–1.1 GPa (MIL confined in 5.39 nm pores). This transformation is also accompanied by γ_ip_C_4,5_H band broadening. Continued pressurization to 2.5 GPa drives MIL bands to higher frequencies. All of these changes are consistent with the spectroscopic changes associated with phase transformation in unconfined [C_2_mim]FeCl_4_. Unfortunately, the SiO_2_ bands obscure γC_2_H in systems with larger pore diameters. In spite of this limitation, we can see an increase in γ_ip_C_4,5_H band center frequency between 0.7 and 1.1 GPa. Evidently, confinement increases the pressure required to solidify the IL. While this effect is expected to depend on the pore diameter, the pressure resolution of our experiment prevents us from establishing a quantitative relationship between pore size and solidification pressure.

## 3. Conclusions

Confining [C_2_mim]FeCl_4_ within SBA-15 mesoporous silica affects the phase behavior, ion transport, and magnetic properties of the material. The reduction in melting point temperature is well-described by the Gibbs–Thomson equation if a 1.60-nm non-freezable layer is postulated to exist along the surface of the pore wall. These observations are in line with the well-established effect of confinement on ambient-pressure phase transition temperatures. Less is known about how confinement affects transition boundaries along the pressure axis of a phase diagram. Our spectroscopic data reveal an increase in isothermal solidification pressure when [C_2_mim]FeCl_4_ is placed inside SBA-15 mesopores. We anticipate other ionic liquids to exhibit similar pressure responses to confinement, and pressure-dependent infrared spectroscopy may provide fertile ground for exploring those systems.

The ionic conductivity of [C_2_mim]FeCl_4_ is lower than the bulk value when it is confined. This suggests the influence of the interfacial regime, where IL dynamics are typically slowed relative to bulk values, extends far into the pores. This is not surprising given the large non-freezable interfacial shell found in the thermal experiments. It is possible the amount of bulk-like liquid available in the pore center for fast ion conduction is relatively small. Therefore, ionic conductivities appear to be largely dominated by surface effects in this system.

Placing the [C_2_mim]FeCl_4_ in a confined geometry also influences the magnetic properties of the ionic liquid. The suppression of the Néel point in the magnetic susceptibility data demonstrates the profound influence confinement has on the long-range antiferromagnetic ordering of [C_2_mim]FeCl_4_. This is possibly related to the MIL’s inability to form large crystalline domains when it is placed inside small-diameter mesopores. Regardless, confinement offers another approach for manipulating the properties of magnetoresponsive ionic liquids beyond the known effects of temperature and pressure.

## 4. Materials and Methods

### 4.1. Sample Preparation

1-Ethyl-3-methylimidazolium tetrachloroferrate(III), [C_2_mim]FeCl_4_, was synthesized from an equimolar mixture of 1-ethyl-3-methylimidazolium chloride (Iolitech, Heilbronn, Germany) and iron(III) chloride (Sigma-Aldrich, St. Louis, Missouri, USA). SBA-15 mesoporous silicas with nominal pore sizes of 12 (sample 1), 10 (sample 2), and 8 nm (sample 3) were purchased from Sigma-Aldrich (St. Louis, Missouri, USA). The mesoporous silicas were heated at 155 °C for 18 h prior to use. Confinement was achieved by mixing appropriate masses of dried silica powder with ionic liquid at 40% [C_2_mim]FeCl_4_ mass and allowing the mixture to equilibrate. When thoroughly mixed, the formerly white SBA-15 has a light-yellow coloration from the MIL. This mass ratio was selected to eliminate unconfined [C_2_mim]FeCl_4_ melting signals in the differential scanning calorimetric (DSC) traces. Higher mass percentages of ionic liquid produced thermal signatures of unconfined MIL.

### 4.2. N_2_ Adsorption Isotherms

The pore structure of the SBA-15 samples was characterized by N_2_ physisorption with a Micromeritics TriStarII 3020 system. Samples were degassed at 90 °C (1 h) and then 150 °C (1 h) under N_2_ flow. Surface areas and pore size distributions were calculated with the Brunauer–Emmett–Teller (BET) and Barrett–Joyner–Halenda (BJH) methods, respectively.

### 4.3. Thermal Characterization 

Melting points of pure and confined [C_2_mim]FeCl_4_ were recorded with a Mettler DSC 1 differential scanning calorimeter. Approximately 10 mg of composite sample was packed in 40-μL Al crucibles and hermetically sealed. Samples were then cooled from 298 to 173 K and then reheated to 298 K with a 10 K min^−1^ scan rate. The ambient pressure during thermal analysis was 98.43 ± 0.16 kPa. The DSC has a ceramic FRS5 sensor containing 56 AuPd thermocouples. The uncertainty in the temperature measurements was ±0.3 K.

### 4.4. Magnetic Property Assessment

Magnetic moments of confined [C_2_mim]FeCl_4_ were measured with a Quantum Design PPMS DynaCool. Samples were cooled to 2 K at a 10 K min^−1^ rate under zero-field conditions. Samples were then subjected to a 1000 Oe field and warmed to 300 K at a rate of 1 K min^−1^. Molar magnetic susceptibilities $\chi_{\mathrm{mol}}$ in emu mol^−1^ Oe^−1^ were computed from the resulting data. Magnetization experiments were conducted on zero-field-cooled composite samples at 2 K with a sweep rate of 100 Oe s^−1^ between ±80 kOe.

### 4.5. Impedance Spectroscopy

Impedance measurements were conducted with a PAR Parstat 2263 frequency response analyzer. Samples were sandwiched between stainless steel blocking electrodes in a parallel plate capacitor configuration. A 10-mV oscillating potential was applied to the samples, and the frequency was swept from 100 mHz to 1 MHz. Temperature regulation between 253 and 303 K was achieved with a Teney T2RC chamber (±0.1 K).

### 4.6. Vibrational Spectroscopy

Mid- and far-infrared spectra were measured at ambient temperature and pressure with Bruker Alpha and Nicolet 6700 FT-IR spectrometers, respectively. A small quantity of each sample was deposited on a diamond attenuated total reflection (ATR) crystal and obtained between 4000 and 400 cm^−1^ (mid-IR) and 700 and 50 cm^−1^ (far-IR). In both cases, spectra were collected with a resolution of 1 cm^−1^.

High-pressure mid-infrared experiments were performed using a Merril–Bassett-type diamond anvil cell (DAC) with two type IIa diamond anvils. The sample was put into a 0.3 mm hole of the inconel gasket. We employed calcium fluoride as a pressure transmitting medium. To remove the absorption of diamonds, DAC absorption was measured first and subtracted from those of the samples.

Raman spectra were acquired with an NXR 9610 FT-Raman spectrometer under ambient pressure and temperature. Materials were housed in quartz NMR tubes and analyzed with a 976 nm excitation laser (power = 1 W) operating in a 180° backscattering geometry. The spectral resolution was set to 4 cm^−1^. 

## Figures and Tables

**Table 1:** Geometric properties for the mesoporous silicas. BJH pore diameters are measured from the desorption branch of the N_2_ isotherm, and BJH pore volumes assume a cylindrical pore shape.

Silica Sample	BET Surface Area (m^2^/g)	BJH Pore Diameter (nm)	BJH Pore Volume (cm^3^/g)
1	500.85	8.36	1.26
2	549.06	7.36	1.03
3	738.90	5.39	0.67

**Table 2:** Melting points and enthalpies of fusion for unconfined and confined [C_2_mim]FeCl_4_.

Sample	Pore Diameter (nm)	Melting Point (K)	ΔH (J/g of [C_2_mim]FeCl_4_)
Unconfined	∞	287.5	63.5
Silica 1	8.36	249.4	21.8
Silica 2	7.36	247.8	21.8
Silica 3	5.39	235.4	11.5

## Data Availability

All data generated are made available in this paper and associated supplemental information.

## Supplementary Materials

The following supporting information can be downloaded at: https://www.mdpi.com/article/10.3390/molecules27175591/s1, Figure S1: Thermal data for [C_2_mim]FeCl_4_ in SBA-15 mesoporous silica; Figure S2: Drop shape of [C_2_mim]FeCl_4_ on SiO_2_; Figure S3: Mid-IR spectra of [C_2_mim]FeCl_4_ in SBA-15 mesoporous silica; Figure S4: Far-IR spectra of [C_2_mim]FeCl_4_ in SBA-15 mesoporous silica.

## Sample Availability

Samples of the compounds are available from the authors.
