# Supplementary material for: Confinement Effects on the Magnetic Ionic Liquid 1-Ethyl-3-methylimidazolium Tetrachloroferrate(III)
Source: Molecules. 2022 Aug 30;27(17):5591. doi: 10.3390/molecules27175591 (PMC9458203; doi:10.3390/molecules27175591)
Supplement: Supplementary file 1 [file molecules-27-05591-s001.zip › molecules-1847589-supplementary.pdf]

molecules

MDPI

Supplementary Materials

Confinement Effects on the Magnetic Ionic Liquid 1-Ethyl-3-Methylimidazolium Tetrachloroferrate(III)

Christopher M. Burba [1,*] and Hai-Chou Chang [2,*]

1 Department of Natural Sciences, Northeastern State University, Tahlequah, OK, USA 74464
2 Department of Chemistry, National Dong Hwa University, Shoufeng, Hualien 974, Taiwan
* Correspondence: burba@nsuok.edu (C.M.B.); hcchang@gms.ndhu.edu.tw (H.-C.C.); Tel.: +1-918-444-5777 (C.M.B.); +886-3-8903585 (H.-C.C.)

Thermal Data for [C_2mim]$FeCl_4$ in SBA-15 Mesoporous Silica

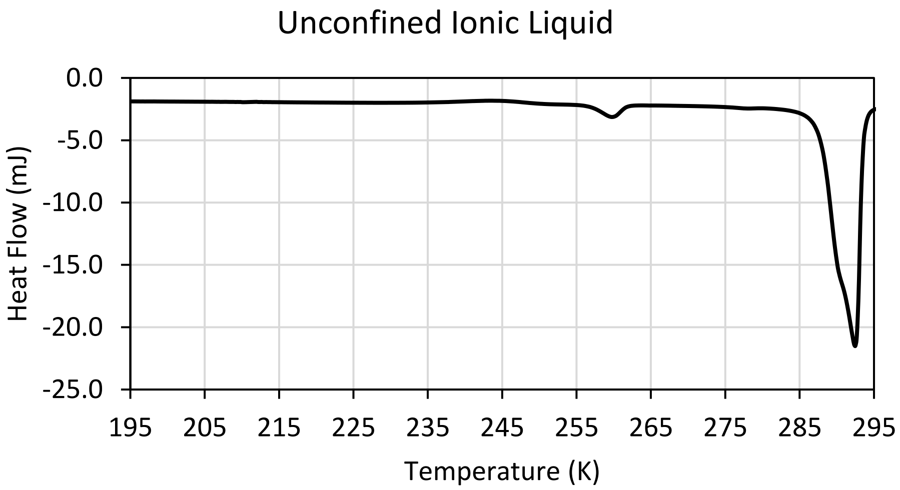

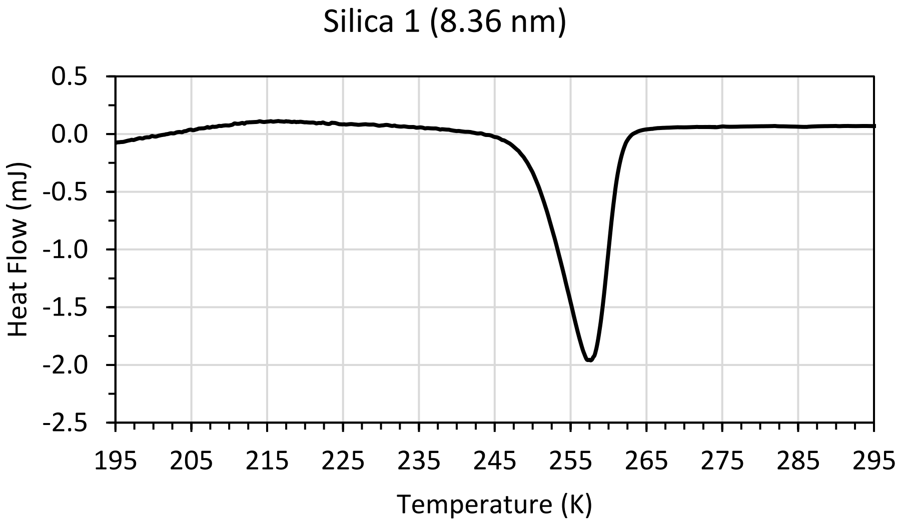

Figure S1. DSC traces of unconfined [C_2mim]$FeCl_4$ and confined [C_2mim]$FeCl_4$ in mesoporous SBA-15 silicas of various diameters. The atmospheric pressure is 98.43 ± 0.16 kPa. Endothermic transitions point down. (Continued on next page).

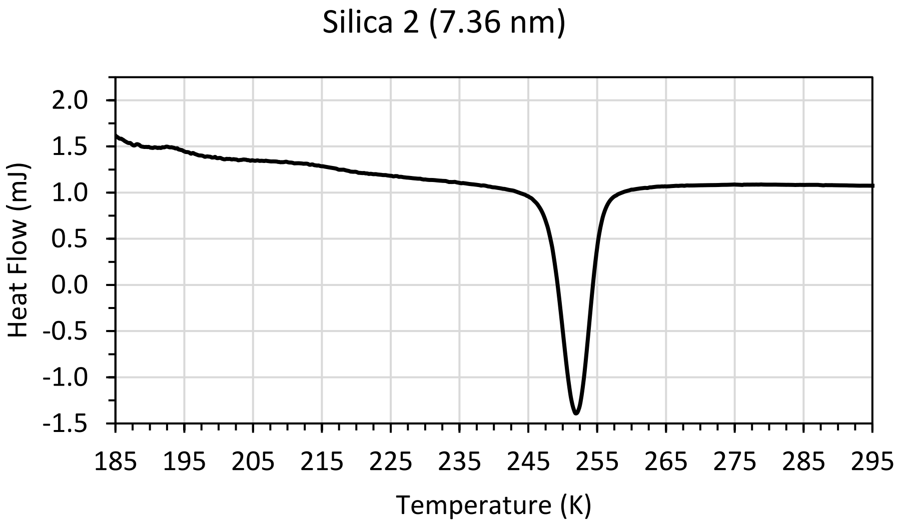

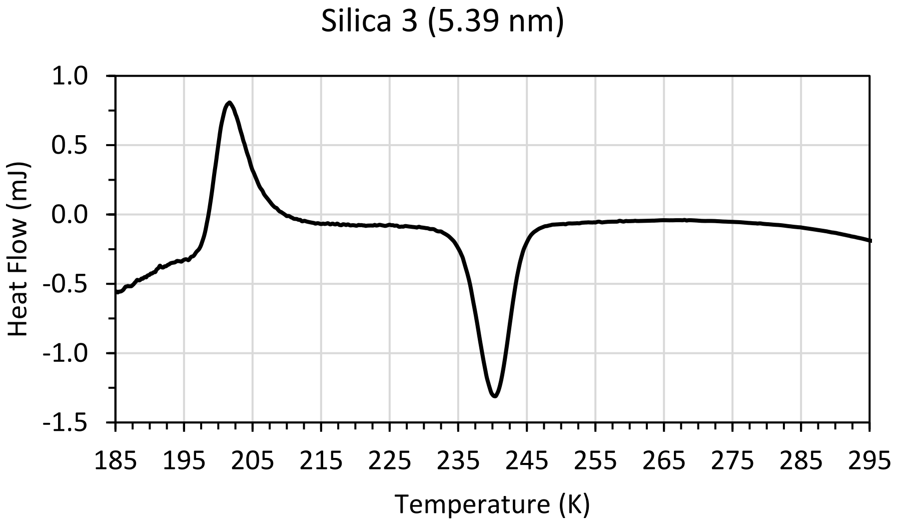

Figure S1. (continued) DSC traces of unconfined [C_2mim]$FeCl_4$ and confined [C_2mim]$FeCl_4$ in mesoporous SBA-15 silicas of various diameters. The atmospheric pressure is 98.43 ± 0.16 kPa. Endothermic transitions point down.

Drop Shape of $[C_2mim]FeCl_4$ on SiO_2

The $[C_2mim]FeCl_4$ contact angle on SiO_2 is measured by placing a small drop of IL on a glass slide. Images are then taken with a 20× macrolens attached to a 12 MP camera (f/1.8 aperture). The image analysis was subsequently performed through ImageJ v 1.8.0 on seven individual drops.

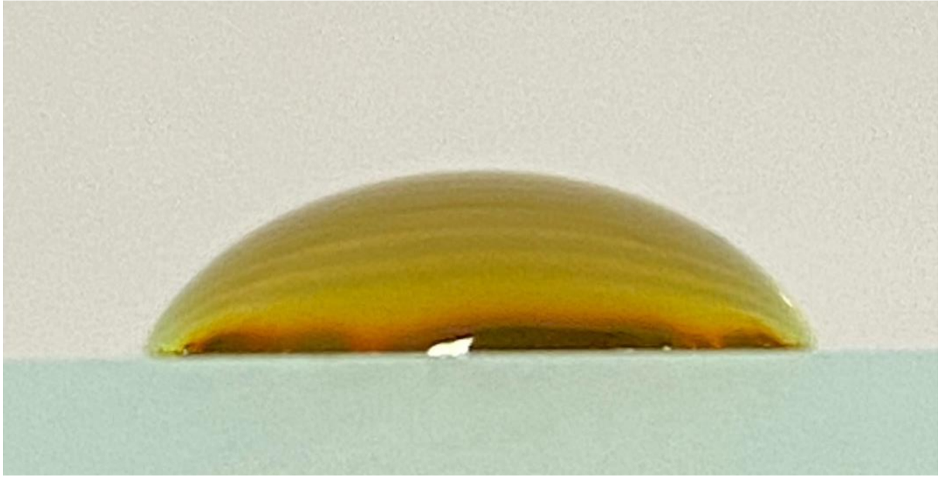

Figure S2. Representative image used to measure the $[C_2mim]FeCl_4$ contact angle on glass slide.

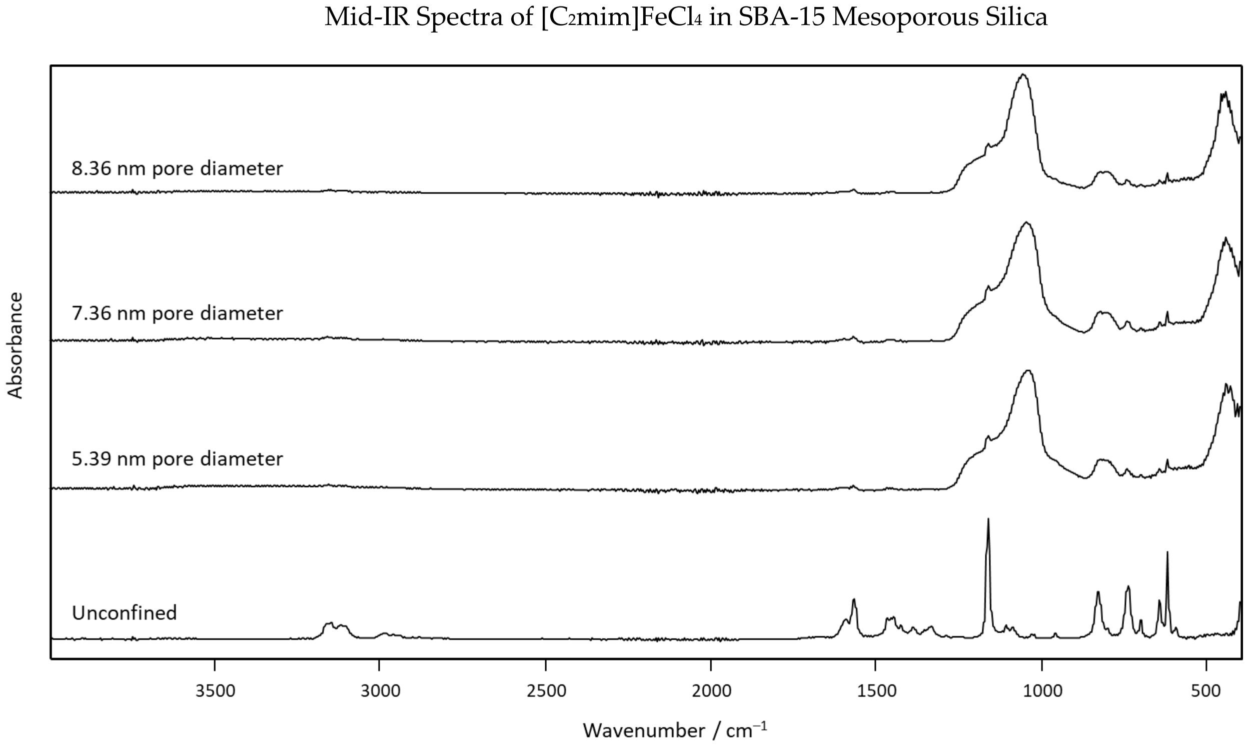

Figure S3. Mid-IR spectra of unconfined [C_2mim]$FeCl_4$ and confined [C_2mim]$FeCl_4$ in mesoporous SBA-15 silicas.

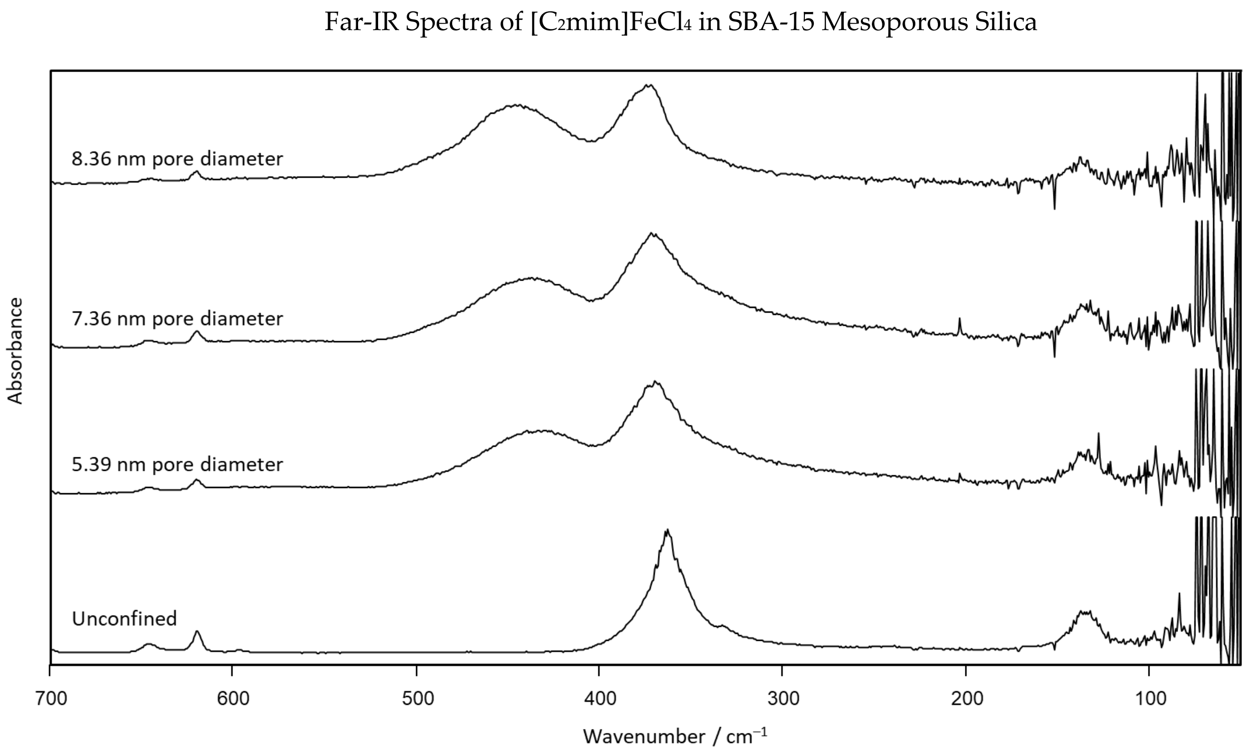

Figure S4. Far-IR spectra of unconfined $[C_2mim]FeCl_4$ and confined $[C_2mim]FeCl_4$ in mesoporous SBA-15 silicas.
